# Systematic evaluation of the methodology of randomized controlled trials of anticoagulation in patients with cancer

**DOI:** 10.1186/1471-2407-13-76

**Published:** 2013-02-14

**Authors:** Gabriel Rada, Holger J Schünemann, Nawman Labedi, Pierre El-Hachem, Victor F Kairouz, Elie A Akl

**Affiliations:** 1 Evidence Based Health Care Program, Faculty of Medicine, Pontificia Universidad Católica de Chile, Santiago, Chile; 2 Department of Clinical Epidemiology and Biostatistics, McMaster University, Hamilton, Canada; 3 Department of Medicine, McMaster University, Hamilton, Canada; 4 Department of Medicine, State University of New York at Buffalo, ECMC-DKM 216,462 Grider St., Buffalo, NY, 14215, USA; 5 School of Medicine, University of Balamand, Beirut, Lebanon; 6 Clinical Research Institute and Department of Internal Medicine, American University of Beirut, Beirut, Lebanon

## Abstract

**Background:**

Randomized controlled trials (RCTs) that are inappropriately designed or executed may provide biased findings and mislead clinical practice. In view of recent interest in the treatment and prevention of thrombotic complications in cancer patients we evaluated the characteristics, risk of bias and their time trends in RCTs of anticoagulation in patients with cancer.

**Methods:**

We conducted a comprehensive search, including a search of four electronic databases (MEDLINE, EMBASE, ISI the Web of Science, and CENTRAL) up to February 2010. We included RCTs in which the intervention and/or comparison consisted of: vitamin K antagonists, unfractionated heparin (UFH), low molecular weight heparin (LMWH), direct thrombin inhibitors or fondaparinux. We performed descriptive analyses and assessed the association between the variables of interest and the year of publication.

**Results:**

We included 67 RCTs with 24,071 participants. In twenty one trials (31%) DVT diagnosis was triggered by clinical suspicion; the remaining trials either screened for DVT or were unclear about their approach. 41 (61%), 22 (33%), and 11 (16%) trials respectively reported on major bleeding, minor bleeding, and thrombocytopenia. The percentages of trials satisfying risk of bias criteria were: adequate sequence generation (85%), adequate allocation concealment (61%), participants’ blinding (39%), data collectors’ blinding (44%), providers’ blinding (41%), outcome assessors’ blinding (75%), data analysts’ blinding (15%), intention to treat analysis (57%), no selective outcome reporting (12%), no stopping early for benefit (97%). The mean follow-up rate was 96%. Adequate allocation concealment and the reporting of intention to treat analysis were the only two quality criteria that improved over time.

**Conclusions:**

Many RCTs of anticoagulation in patients with cancer appear to use insufficiently rigorous outcome assessment methods and to have deficiencies in key methodological features. It is not clear whether this reflects a problem in the design, conduct or the reporting of these trials, or both. Future trials should avoid the shortcomings described in this article.

## Background

Venous thromboembolism (VTE), which includes deep vein thrombosis (DVT) and pulmonary embolism (PE), is a common disorder in patients with cancer [1]. It is an important cause of morbidity and one of the leading causes of death in that population [2].

Anticoagulants are used for treatment of VTE and for thromboprophylaxis in high risk conditions such as surgery or the presence of an indwelling central venous catheter [3-6]. A survival benefit from anticoagulants as a result of VTE prevention and a possible direct antitumor effect has also been explored [7,8].

High-quality randomized controlled trials (RCTs) are the preferred method to establish the effects on efficacy and safety outcomes, since they minimize systematic error (bias). However, the reliability of their results depends on the extent to which potential sources of bias have been avoided [9]. Both the Cochrane Collaboration and the GRADE working group have advanced the methods to define which criteria should be evaluated in order to judge the risk of bias of a trial or a body of trials [9,10]. Poorly designed and/or conducted RCTs may lead to biased results, mislead clinical practice and adversely affect patients’ outcomes. Unfortunately, overwhelming evidence shows the quality of RCTs is not optimal [11].

A few studies assessing the quality or the reporting of RCTs in patients with cancer have concluded that the quality of reporting is low, and some suggest that it is lower than in other areas [12-16]. However, we have identified no study focusing on trials of anticoagulation in patients with cancer.

The objective of this study is to systematically describe the characteristics, the risk of bias and their time trends in RCTs of anticoagulation in patients with cancer [3-8].

## Methods

The study sample consists of all trials included in a series of six Cochrane systematic reviews of anticoagulation in patients with cancer [3-8]. The series covered the majority of topics for which RCTs were conducted in this field: parenteral anticoagulation for survival benefit (VTE thromboprophylaxis trials in ambulatory patients with cancer), oral anticoagulation for survival benefit, central venous catheters thromboprophylaxis, perioperative thromboprophylaxis, initial anticoagulation treatment of VTE, and long term anticoagulation treatment of VTE. A common search was conducted for all the reviews.

### Eligibility criteria

Inclusion criteria for this study were the following: studies describing random allocation; participants with cancer of any type or stage; intervention and/or comparison consisting of vitamin K antagonists, unfractionated heparin (UFH), low molecular weight heparin (LMWH), ximelagatran, dabigatran, rivaroxaban, apixaban, or fondaparinux. We have included the details of the eligibility criteria for each of the six Cochrane systematic reviews in Additional file 1.

### Search strategy

We electronically searched the following databases: the Cochrane Central Register of Controlled Trials (CENTRAL) (The Cochrane Library 2010, Issue 1), MEDLINE (1966 to February 2010; accessed via Ovid), EMBASE (1980 to February 2010; accessed via Ovid) and ISI Web of Science (February 2010). The search strategies combined free text and controlled vocabulary terms for cancer and anticoagulants, and a sensitive search strategy to retrieve randomized clinical trials (available from the authors). We have included the details of these electronic search strategies in Additional file 2.

In addition, we hand searched the conference proceedings of the American Society of Clinical Oncology (ASCO, starting with its first volume, 1982) and of the American Society of Hematology (ASH, starting with its 2003 issue); we screened the reference lists of included studies and of other relevant systematic reviews; and we used the related article feature in PubMed. We did not use language restrictions. Detailed eligibility criteria for each systematic review are reported in Additional file 1[3-8].

### Selection of studies

Two reviewers independently screened the titles and abstracts of identified citations for potential eligibility. Then, two reviewers independently screened the full text of articles potentially eligible using a standardized form. They resolved disagreements by discussion or by consulting a third reviewer.

### Data extraction

Two reviewers independently extracted the data in duplicate from each study. They used a standardized form and resolved their disagreements by discussion or by consulting a third author. We considered all available reports for any particular trial to assess methodological quality. We attempted to contact authors for missing or unclearly reported data.

We extracted information about: publication year, language, funding (governmental, for profit, not for profit), population studied, number of participants, interventions, outcomes and assessment methods of DVT and PE (screening and diagnosis). Additionally, we evaluated the following risk of bias criteria: adequacy of sequence generation; adequacy of allocation concealment; blinding of participants, providers, data collectors, outcome assessors and data analysts; intention to treat analysis; absence of selective outcome reporting; no early stopping for benefit; and percentage of follow-up. For questions about blinding we further categorized unclearly reported data into ‘probably yes’ and ‘probably no’ using validated specific instructions (see Figure 1 in Akl et al., Journal of Clinical Epidemiology) [17].

**Figure 1:**
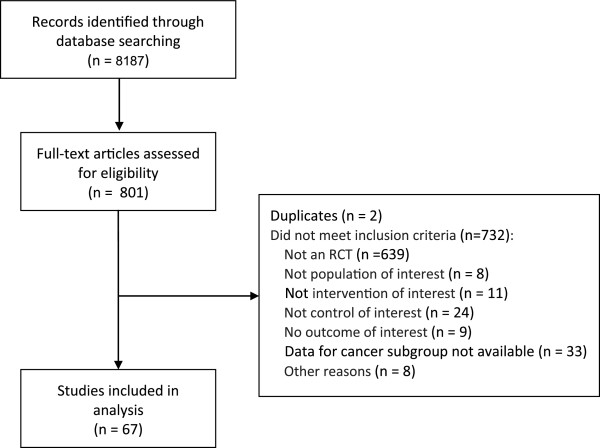
Study flow.

### Statistical analyses

We used frequency percentage, median and interquartile range to describe the trials’ general characteristics and risk of bias criteria. We also assessed temporal trends for funding, number of participants, assessment methods, and outcome reporting.

Given that number of trials was not equally distributed in time, with a small sample in the 80s, we assessed the association between methodological quality criteria and the year of publication using chi-square test for linear trend. We used 9-year intervals in order to cover the 27 year interval since the publication of the oldest trial identified.We assessed the association between industry funding and risk of bias criteria using chi-square test. Also, we performed regression analyses considering each of the risk of bias criteria as the dependent variable and year, sample size, review topic and funding as independent variables.

We performed all analyses using SPSS (version 17.0).

## Results

### Search results

Figure 1 shows the study flow with reasons for exclusions. The search identified 8187references, of which we included sixty-seven unique RCTs, including a total of 24071 participants.

### General characteristics

**Figure 2:**
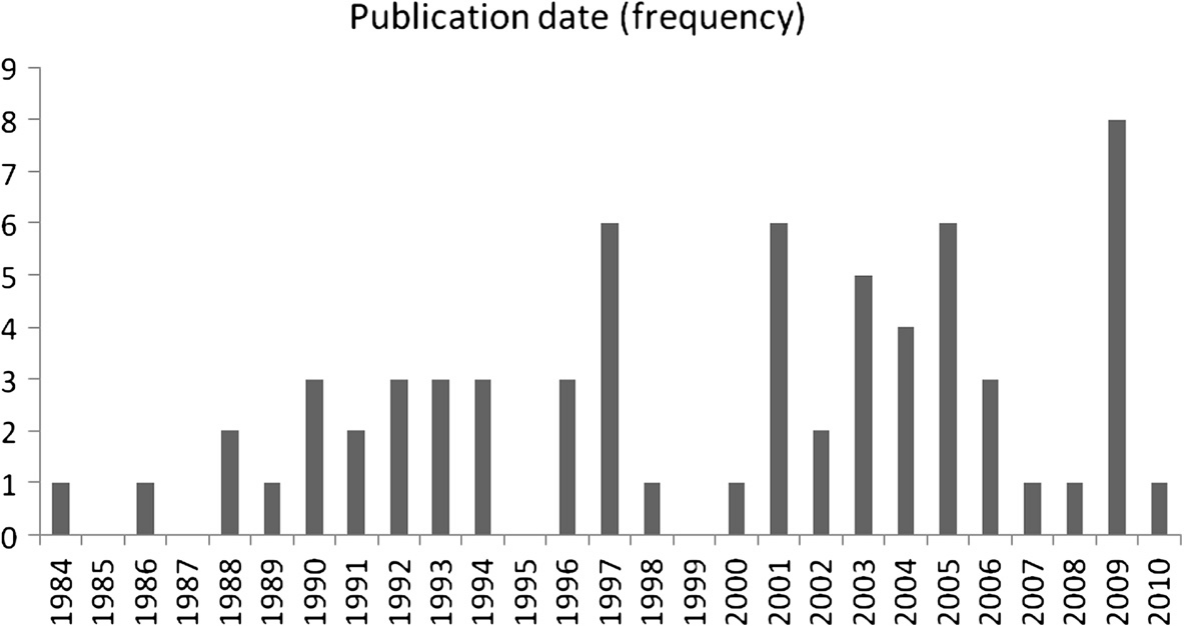
Time trend of the number of randomized controlled trials evaluating anticoagulation in cancer.

The publication year was in the 1980s, 1990s, and 2000s for 7%, 36% and 57% of trials respectively. Figure 2 shows the distribution by the year of publication from 1984 to 2010.

Tables 1 and 2 provide the general characteristics and the methods of assessment of VTE in the studies.

**Table 1:** General characteristics of randomized controlled trials

	**Overall, n (%) N=67**
**Funding source**	
governmental	12 (18)
private not for profit	3 (4)
private for profit	41 (61)
Private for profit plus other source	4 (6)
Not funded	3 (4)
Not reported	14 (21)
**Publication language**	
English	67 (100)
**Sample size**	
Median (Q1-Q3)	156 (74-355)
n <200	39 (58)
n ≥200	28 (42)
**Type of cancer**	
Participants with different cancer types	47 (70)
Cancer type not reported	5 (7)
All participants with same cancer type	15 (22)
Lung	5 (33)
Colorectal	2 (13)
Gynecologic	2 (13)
Prostate	1 (7)
Breast	1 (7)
Pancreatic	1 (7)
Hematological	2 (13)
Hematological and solid tumors	1 (6)
Cancer as subgroup of the study	27 (40)
**Intervention**	
LMWH	53 (79)
UFH	33 (49)
Vitamin K antagonist	20 (30)
Direct thrombin inhibitors	5 (7)
Fondaparinux	3 (4)
**Comparator**	
Active treatment	47 (70)
Placebo	9 (13)
No treatment	11 (17)

LMWH=Low molecular weight heparin, UFH=Unfractioned Heparin, VTE=Venous thromboembolism, PE=Pulmonary embolism, DVT=Deep vein thrombosis.

**Table 2:** Assessment of VTE

	**Overall, n (%) N=67**
**Screening for DVT**	
Venography	31 (46)
125I-Fibrinogen-uptake test	14 (45)
Impedance plethysmography	7 (23)
Doppler Ultrasound	4 (13)
CT scan	5 (16)
No screening reported	1 (3)
	36 (54)
**Screening for PE**	
Ventilation/Perfusion Scan	6 (9)
Pulmonary Angiography	6 (100)
No screening reported	1 (15)
	61 (91)
**Diagnosis of clinically suspected DVT**	
Venography	51 (76)
125I-Fibrinogen-uptake test	44 (86)
Impedance plethysmography	4 (8)
Doppler Ultrasound	4 (8)
CT scan	30 (59)
Not reported	2 (4)
	16 (24)
**Diagnosis of clinically suspected PE**	
Ventilation/Perfusion Scan	44 (66)
CT of Thorax	40 (91)
Pulmonary Angiography	17 (39)
Autopsy	33 (75)
Not reported	12 (27)
	23 (34)
**Outcomes reported**	
Death	53 (79)
VTE	25 (37)
PE	31 (46)
DVT	38 (57)
Major bleeding	41 (61)
Minor bleeding	22 (33)
Thrombocytopenia	11 (16)

CT= Computed tomography, VTE=Venous thromboembolism, PE=Pulmonary embolism, DVT=Deep vein thrombosis.

English was the language of publication in 100% of studies. The more common source of funding was private for profit (n=41; 61%). Fourteen studies (21%) did not report the funding source. No specific type of funding has increased or decreased significantly over time. The median sample size was 156 participants (Q1-Q3: 74-355), having increased over time from 100 participants per trial (Q1-Q3: 52-352) before 1993 to 186 after 2001 (Q1-Q3: 88-385) (p<0.001).

Forty seven studies (70%) assessed participants with different types of cancer, while 15 (22%) included only one type (lung, colorectal, gynecologic, prostate, breast, pancreatic, hematological, hematological and solid tumors).

The most frequently studied medication class was low molecular weight heparin in 53 studies (79%). Nine studies evaluated intervention against a placebo (13%). The comparison with placebo was associated with later years of publication (p=0.009).

Studies reported data for death, venous thromboembolism, pulmonary embolism, and DVT in 53 (79%), 25 (37%), 31 (46%), and 38 (57%) of cases respectively. Only 21 studies (31%) reported data on diagnosis triggered by clinical suspicion; the rest reported data on DVT screening or did not report data. Assessments of venous thromboembolism and of major bleeding were associated with more recent years of publication (p=0.002 and 0.001 respectively).

The number of trials reporting some method for the screening of DVT decreased from 69 % in the first period to 26% in the later period (p=0.005).

The use of the 125I-fibrinogen-uptake test, a test with inferior diagnostic properties, was associated with earlier publication (p<0.001). Use of Ventilation/Perfusion scan as a screening tool was associated with earlier publication (p=0.01), and use of Doppler with diagnostic purpose was associated with recent publication (p<0.001).

### Reported risk of bias of RCTs

Table 3 describes the risk of bias of the included RCTs.

**Table 3:** Risk of bias in randomized controlled trials

	**Overall, n (%) N=67**
**Sequence generation**	
Adequate	57 (85)
Not clear	10 (15)
Inadequate	0 (0)
**Allocation concealment**	
Adequate	41 (61)
Inadequate	3 (5)
Not clear	23 (34)
**Participants blinded**	
Definitely yes	5 (8)
Probably yes	21 (31)
Probably no	13 (19)
Definitely no	28 (42)
**Caregiver blinded**	
Definitely yes	5 (8)
Probably yes	22 (33)
Probably no	13 (19)
Definitely no	27 (40)
**Data collector blinded**	
Definitely yes	3 (5)
Probably yes	26 (39)
Probably no	13 (19)
Definitely no	25 (37)
**Outcome assessor blinded**	
Definitely yes	34 (51)
Probably yes	16 (24)
Probably no	7 (10)
Definitely no	10 (15)
**Data analyst blinded**	
Definitely yes	7 (10)
Probably yes	3 (5)
Probably no	37 (55)
Definitely no	20 (30)
**Intention to treat**	
Yes	38 (57)
Unclear	15 (22)
No	14 (21)
**Free of selective reporting of**	
**outcomes**	
Adequate	59 (88)
Not clear	5 (7)
Inadequate	3 (5)
**Stopped early**	
Total	7 (10)
For benefit	2 (3)
For harm	1 (2)
For insufficient accrual	4 (6)
Not reported	0 (0)
**Follow up**	
Mean percentage (range)	96% (57-100)

The reporting of only two risk of bias criteria has significantly improved over time. Adequate allocation concealment increased from 39% before 1993 to 71% after 2001 (p=0.046), and the reporting of an intention to treat analysis increased from 31% to 77% (p=0.033). The remaining risk of bias criteria did not significantly change with the year of publication.

In the bivariable analysis, industry funding was significantly associated with absence of caregiver blinding (p=0.043) and use of intention to treat analysis (p=0.01). Regression analysis showed that intention to treat was significantly associated with type of funding (OR 0.172; 95% CI 0.046-0.648). None of the other risk of bias criteria was significantly associated with type of funding, review topic, year, or sample size.

## Discussion

Our study describes the characteristics, the risk of bias and the related time trends in randomized trials of anticoagulation in cancer. We used a comprehensive search strategy and a systematic methodology for selecting studies and abstracting data.

We found that the majority of RCTs of anticoagulation in patients with cancer appear to use insufficiently rigorous outcome assessment methods and to have deficiencies in key methodological features. Specifically, included studies had low rates of blinding of participants and caregivers, and intention to treat analysis. Our findings likely reflect a problem in the design, conduct and the reporting of these trials.

It has to be acknowledged it might be particularly challenging for trials of anticoagulation in the cancer setting, relative to other types of interventions in other populations, to adhere to some of the methods employed to reduce the risk of bias. For example, clinicians caring for patients with active cancer might be reluctant to accept to be blinded given their patients are already at increased risk of bleeding, frequently undergo invasive procedures, and might have chemotherapy-induce thrombocytopenia.

Our findings are consistent with those of several methodological studies in cancer [12-16,18-23]. When improvement over time has been explored, they have also found advances in some criteria, but at a rate slower than expected [16,18]. One study suggested that reporting is the main problem in trials of radiation therapy in oncology [13].

Given standard treatments have been established by previous studies as effective and safe, 70% of included trials had an active comparator (as opposed to placebo or no intervention). In our assessment of risk of bias, we did not consider the use of an active comparator as inappropriate. Rather, we focused on whether those involved in the trial were blinded to allocation. While blinding of providers and participants is feasible in these cases, it is challenging in the situations where different routes of administration are used, and dosage adjustments are required. However, given the risk of bias associated with a lack of blinding, this could be overcome with double dummy designs, sham-adapted dosages and several other techniques [24]. Considering that lack of blinding can overestimate treatment effect estimates, it is important to make every effort to ensure blinding in studies [25]. Failure to report intention to treat analysis can incorporate systematic and unpredictable bias in a trial [26]. Other types of analysis (i.e. per protocol analysis) should not be presented in isolation. It is surprising that only the reporting of adequate allocation concealment and of intention to treat analysis significantly changed over time. However, and especially for the latter, there is ample room for improvement.

Only 31% of studies reported data on diagnosis of deep vein thrombosis triggered by clinical suspicion and the rest reported data on DVT screening. However, estimating absolute symptomatic events from studies that included screening may be misleading [27]. If screening is positive, patients will typically receive anticoagulation. If they had not received anticoagulation, some of them would likely have developed symptomatic VTE. This would underestimate the number of symptomatic VTE and the benefit of any intervention that reduces VTE. Bias in the opposite direction is also possible. A positive screening test may lead to interpreting any minor symptom, that otherwise might not have warranted investigation, as a symptomatic VTE. This would overestimate the number of symptomatic VTE, and the benefit of any intervention that reduces VTE. Others might argue that screening-identified asymptomatic may be more informative than misleading. Indeed, there is evidence that incidentally diagnosed PEs have similar prognostic value on survival as symptomatic PEs [28,29]. Moreover, false positive results should be minimized in the hands of experienced imaging centers thus avoiding some of the above concerns. More research is needed to shed more light on this controversial area.

Interestingly, 20% of trials do not report the source of funding. Of those who report, 3 of every 4 trials are completely or partially funded by private for profit sources. Industry funded trials were more likely to report intention to treat analysis, and less likely to report caregiver blinding. It is well known that industry sponsored trials favor products made by the company funding the research, but one of the more likely explanations is not because a higher risk of bias but through the selection of an inappropriate comparator to the product being investigated [30,31].

We have found that the topic of the systematic review not to be associated with risk of bias. While this might reflect the absence of such association, it might be simply due to lack of statistical power given the relatively small number of studies included in the analysis.

### Implications for future trial conduct and reporting

Existing guidelines of reporting are widely available. Trialists of anticoagulation in patients with cancer should design more rigorous trials and transparently report their methods and findings.

### Implications for future methodological research

A recent systematic review found 177 studies evaluating the methodological quality of RCTs. While this type of studies is increasing, they suffer from a heterogeneity of criteria used and lack of clear definitions. Our study fulfills most of the criteria of quality of reporting proposed by these authors [32]. In the absence of other specific guidelines for the reporting of this type of methodological reviews, these criteria could inform future work.

## Conclusions

Many RCTs of anticoagulation in patients with cancer appear to use insufficiently rigorous outcome assessment methods and to have deficiencies in key methodological features. It is not clear whether this reflects a problem in the design, conduct or the reporting of these trials, or both. Future trials should avoid the shortcomings described in this article.

## Competing interests

The authors declare that they have no competing interests.

## Authors’ contributions

GR performed the statistical analysis and drafted the manuscript. HS participated in the design of the study and provided methodological expertise. NL, PE and VK extracted the data from individual studies. EA conceived the study, participated in its design and coordination, extracted the data from studies and helped to draft the manuscript. All authors read and approved the final manuscript.

## Pre-publication history

The pre-publication history for this paper can be accessed here:

http://www.biomedcentral.com/1471-2407/13/76/prepub

## Supplementary Material

Additional file 1 Eligibility criteria for individual reviews. Click here for file

Additional file 2 Search strategies for electronic databases. Click here for file
