# Supplementary material for: Systematic evaluation of the methodology of randomized controlled trials of anticoagulation in patients with cancer
Source: BMC Cancer. 2013 Feb 14;13:76. doi: 10.1186/1471-2407-13-76 (PMC3579688; doi:10.1186/1471-2407-13-76)
Supplement: Additional file 1 — Eligibility criteria for individual reviews. [file 1471-2407-13-76-S1.docx]

**Appendix 1. Eligibility criteria for individual reviews**

**Review 1. Anticoagulation for the long-term treatment of venous thromboembolism in patients with cancer**

**Types of studies:** Randomized controlled trials (RCTs).

**Types of participants:** Patients with cancer with a confirmed diagnosis of VTE (deep venous thrombosis (DVT) or pulmonary embolism). Patients could have been of any age group (including pediatric patients), with either solid or hematological cancer, at any stage of their cancer and irrespective of the type of cancer therapy.
DVT should have been diagnosed using one the following objective diagnostic tests: venography, 125I-fibrinogen-uptake test, impedance plethysmography or Doppler-ultrasound. Pulmonary embolism should have been diagnosed using one of the following objective diagnostic tests: pulmonary perfusion/ventilation scans, computed tomography or pulmonary angiography).

**Types of interventions:** We included studies comparing long-term treatment with LMWH versus oral anticoagulants (VKA or ximelagatran). There should have been no differences in how the study groups were treated besides the main intervention (e.g. the type of initial anticoagulation). That is studies should have treated patient groups similarly apart from the intervention of interest.

**Types of outcome measures:**

*Primary outcomes:*Survival

*Secondary outcomes:*Symptomatic recurrent DVT; events had to be diagnosed using one of the following objective diagnostic tests: venography, 125I-fibrinogen-uptake test, impedance plethysmography or Doppler-ultrasound
Symptomatic recurrent pulmonary embolism; events had to be diagnosed using one of the following objective diagnostic tests: pulmonary perfusion/ventilation scans, computed tomography, pulmonary angiography or autopsy
Major bleeding
Minor bleeding
Thrombocytopenia
Postphlebitic syndrome
We accepted the definitions of major bleeding, minor bleeding, thrombocytopenia and postphlebitic syndrome of the authors of the original studies as long as they were standardized.

**Review 2. Anticoagulation for the initial treatment of venous thromboembolism in patients with cancer**

**Types of studies:** Randomized controlled trials (RCTs).

**Types of participants:** Patients with cancer and a confirmed diagnosis of VTE (acute deep venous thrombosis or pulmonary embolism). Patients could have been of any age group (including pediatric patients) with either solid or hematological cancer and at any stage of their cancer irrespective of the type of cancer therapy.
To include patients, deep venous thrombosis should have been diagnosed using one the following objective diagnostic tests: venography, 125I-fibrinogen uptake test, impedance plethysmography, or Doppler ultrasound. Pulmonary embolism should have been diagnosed using one the following objective diagnostic tests: pulmonary perfusion or ventilation scans, computed tomography, pulmonary angiography).

**Types of interventions:** We considered comparisons of the following agents used in initial parenteral anticoagulation (typically the first five to 10 days): LMWH, UFH, or fondaparinux. We excluded studies in which thrombolytic therapy (for example streptokinase) was part of the intervention. The protocol should have planned to provide all other co-interventions (for example chemotherapy) similarly.

**Types of outcome measures:**

*Primary outcome:s*All cause mortality

*Secondary outcomes:*Symptomatic recurrent deep venous thrombosis; events had to be diagnosed using one of the following objective diagnostic tests: venography, 125I-fibrinogen uptake test, impedance plethysmography, or Doppler ultrasound
Symptomatic recurrent pulmonary embolism; events had to be diagnosed using one of the following objective diagnostic tests: pulmonary perfusion or ventilation scans, computed tomography, pulmonary angiography or autopsy
Major bleeding
Minor bleeding
Postphlebitic syndrome
Quality of life
Thrombocytopenia
We accepted the authors' definitions of major bleeding, minor bleeding, thrombocytopenia, and postphlebitic syndrome as long as they were standardized.

**Review 3. Anticoagulation for patients with cancer and central venous catheters**

**Types of studies:** Randomized controlled trials (RCTs).

**Types of participants:** Cancer patients with a CVC of any age (including paediatric patients), with either solid or haematological cancer, at any stage of their cancer and irrespective of the type of cancer therapy received. Patients had to have no clinical evidence of VTE at enrolment.

**Types of interventions:** Experimental intervention: parenteral anticoagulants (including UFH, LMWH, and fondaparinux) or oral anticoagulants (including VKA) irrespective of the dose.
Comparison: another anticoagulant, placebo or no intervention.
The protocol should have planned to provide all other co-interventions (e.g. chemotherapy) similarly.

**Types of outcome measures:** The outcome measures did not constitute criteria for including studies.

*Primary outcomes:*All cause mortality.

*Secondary outcomes:*Premature CVC removal.
Catheter-related bacteraemia.
Catheter exit site infection. The diagnosis of catheter-related bacteraemia and catheter exit site infection had to be based on pre-defined criteria. We did not consider catheter colonization.
CVC site DVT. The diagnosis of CVC-thrombosis could have resulted either from screening with the described screening methods or from clinical suspicion with subsequent confirmation by one of the described tests.
Non-CVC site DVT. DVT events had to be diagnosed using an objective diagnostic test such as: venography, 125I-fibrinogen-uptake test, impedance plethysmography, or compression ultrasound.
Pulmonary embolism. PE events had to be diagnosed using an objective diagnostic test such as: pulmonary perfusion/ventilation scan, computed tomography, pulmonary angiography or autopsy.
Major bleeding: we accepted the authors' definitions of minor bleeding.
Minor bleeding; we accepted the authors' definitions of minor bleeding.
Thrombocytopenia: we accepted the authors' definitions.
Heparin induced thrombocytopenia (HIT).
Heparin induced thrombocytopenia with thrombosis (HITT).
Quality of life.

**Review 4. Low molecular weight heparin versus unfractionated heparin for perioperative thromboprophylaxis in patients with cancer**

**Types of studies:** Randomized controlled trials (RCTs).

**Types of participants:** Patients with cancer planned to undergo a surgical intervention

**Types of interventions:** Experimental intervention: Low Molecular Weight Heparin (LMWH)
Comparator intervention: Unfractionated Heparin (UFH).
The protocol should have planned to provide all other co-interventions similarly in the intervention and comparison group.

**Types of outcome measures:** The outcome measures did not constitute criteria for including studies.

*Primary outcomes:*
All cause mortality

*Secondary outcomes:*Symptomatic PE
Symptomatic DVT
Asymptomatic DVT
Bleeding outcomes:
Major bleeding
Minor bleeding
Wound hematoma
Reoperation for bleeding
Thrombocytopenia

**Review 5. Parenteral anticoagulation in patients with cancer who have no therapeutic or prophylactic indication for anticoagulation**

**Types of studies:** Randomized controlled trials.

**Types of participants:** Patients with cancer with no indication for prophylactic anticoagulation (e.g. for acute illness, for central venous line placement, perioperatively) or for therapeutic anticoagulation (e.g. for the treatment of DVT or pulmonary embolism).

**Types of interventions:** Experimental intervention: parenteral anticoagulants such as UFH, LMWH and fondaparinux.
Comparator intervention: placebo or no intervention.
We also considered studies comparing different parenteral anticoagulants. The protocol should have planned to provide all other co-interventions (e.g. chemotherapy) similarly.

**Types of outcome measures:**The outcome measures did not constitute criteria for including studies.

*Primary outcomes:*All-cause mortality; pre-specified at 12 months, 24 months and over the duration of the trial.

*Secondary outcomes:*Symptomatic venous thromboembolism (DVT and/or pulmonary embolism): DVT events had to be diagnosed using an objective diagnostic test such as: venography, 125I-fibrinogen-uptake test, impedance plethysmography or compression ultrasound. Pulmonary embolism events had to be diagnosed using an objective diagnostic test such as: pulmonary perfusion/ventilation scans, computed tomography, pulmonary angiography or autopsy.
Health-related quality of life: had to be measured using a validated tool.
Major bleeding: we accepted the authors' definitions of major bleeding.
Minor bleeding: we accepted the authors' definitions of minor bleeding.
Thrombocytopenia.

**Review 6. Oral anticoagulation in patients with cancer who have no therapeutic or prophylactic indication for anticoagulation**

**Types of studies:** Randomized controlled trials.

**Types of participants:** Patients with cancer with no indication for prophylactic anticoagulation (e.g. for acute illness, for central venous line placement, perioperatively) or for therapeutic anticoagulation (e.g. for the treatment of deep venous thrombosis (DVT) or pulmonary embolism).

**Types of interventions:** Main intervention: vitamin K antagonist or other oral anticoagulants.
Comparison: placebo or no intervention.
We also considered studies comparing different oral anticoagulants. The protocol from original studies should have planned to provide all other co-interventions (e.g. chemotherapy) similarly.

**Types of outcome measures:** The outcome measures did not constitute criteria for eligibility.

*Primary outcomes:*All cause mortality

*Secondary outcomes:*Symptomatic venous thromboembolism (DVT and/or pulmonary embolism): DVT events had to be diagnosed using an objective diagnostic test such as: venography, 125I-fibrinogen-uptake test, impedance plethysmography, or compression ultrasound. Pulmonary embolism events had to be diagnosed using an objective diagnostic test such as: pulmonary perfusion/ventilation scans, computed tomography, pulmonary angiography or autopsy;
Health related quality of life: had to be measured using a validated tool;
Major bleeding: we accepted the authors' definitions of major bleeding;
Minor bleeding; we accepted the authors' definitions of minor bleeding.
